# The prognostic significance of inflammation-immunity-nutrition score on postoperative survival and recurrence in hepatocellular carcinoma patients

**DOI:** 10.3389/fonc.2022.913731

**Published:** 2022-08-09

**Authors:** Yuxin Liang, Zilong Zhang, Deyuan Zhong, Chunyou Lai, Zonglin Dai, Haibo Zou, Tianhang Feng, Jin Shang, Ying Shi, Xiaolun Huang

**Affiliations:** ^1^ Department of Hepatobiliary-Pancreatic Surgery, Cell Transplantation Center, Sichuan Provincial People’s Hospital, University of Electronic Science and Technology of China, Chengdu, China; ^2^ Clinical Immunology Translational Medicine Key Laboratory of Sichuan Province and Organ Transplant Research Institute, Sichuan Provincial People’s Hospital, University of Electronic Science and Technology of China, Chengdu, China; ^3^ Chinese Academy of Sciences Sichuan Translational Medicine Research Hospital, Chengdu, China

**Keywords:** inflammation-immunity-nutrition score, alpha-fetoprotein, hepatocellular carcinoma, prognosis, recurrence

## Abstract

**Background:**

Inflammation, immunity, and nutrition status play important roles in tumorigenesis, progression, and metastasis. This study aimed to evaluate the prognostic value of Inflammation-Immunity-Nutrition Score (IINS) for overall survival (OS) and progression-free survival (PFS) in patients with hepatocellular carcinoma (HCC) undergoing radical surgery.

**Methods:**

A total of 204 HCC patients who met the criteria were included in this retrospective study: 144 in the prediction model and 60 in the validation model. IINS was constructed based on the sum of classification scores of preoperative high-sensitivity C-reactive protein (hsCRP), lymphocyte (LYM), and albumin (ALB). The associations between the IINS group and the clinicopathologic characteristics were analyzed using Pearson’s χ2 test or Fisher’s exact test. Multivariate Cox regression analysis was used to evaluate variables significant on univariate analysis. Kaplan-Meier survival curves were conducted to investigate the prognostic values of IINS, Alpha-fetoprotein (AFP) and IINS-AFP classification. The prognostic performances of all the potential prognostic factors were further compared by receiver operating characteristic (ROC) curve, and time-dependent ROC curve. The internal validation and external validation were used to ensure the credibility of this prediction model.

**Results:**

The patients were divided into low and high IINS groups according to the median of IINS. According to multivariate Cox regression analyses, the Barcelona Clinic Liver Cancer (BCLC) Stage (P=0.003), AFP (P=0.013), and IINS (P=0.028) were independent prognostic factors for OS, and BCLC Stage (P=0.009), microvascular invasion (P=0.030), and IINS (P=0.031) were independent prognostic factors for PFS. High IINS group were associated with significantly worse OS and PFS compared with low IINS group (P<0.001; P=0.004). In terms of clinical prognosis, IINS-AFP classification was good in group I, moderate in group II, and poor in group III. Group I had a longer OS (P<0.001) and PFS (P=0.008) compared with group II and III. ROC analysis revealed that IINS-AFP classification had a better prognostic performance for OS (AUC: 0.767) and PFS (AUC: 0.641) than other predictors, excluding its slightly lower predictive power for PFS than IINS. The time-dependent ROC curves also showed that both IINS (12-month AUC: 0.650; 24-month AUC: 0.670; 36-month AUC: 0.880) and IINS-AFP classification (12-month AUC: 0.720; 24-month AUC: 0.760; 36-month AUC: 0.970) performed well in predicting OS for HCC patients. Furthermore, the internal validation and external validation proved that IINS had good predictive performance, strong internal validity and external applicability, and could be used to establish the prediction model.

**Conclusion:**

Inflammation-immunity-nutrition score could be a powerful clinical prognostic indicator in HCC patients undergoing radical surgery. Furthermore, IINS-AFP classification presents better prognostic performance than IINS or AFP alone, and might serve as a practical guidance to help patients adjust treatment and follow-up strategies to improve future outcomes.

## Introduction

Hepatocellular carcinoma (HCC), one of the most common malignant tumors, is also the leading cause of cancer-related death worldwide. Its global incidence continues to rise and might surpass an annual incidence of 1 million cases (1–3). For the treatment of patients with early-stage HCC, surgical resection, liver transplantation, and radiofrequency ablation are all first-line radical treatments (4, 5). Moreover, transarterial chemoembolization (TACE), targeted therapy, and immunotherapy are currently considered the three most promising approaches for patients with advanced HCC (3, 6–8). However, the high recurrence rate of HCC after curative surgery limits its therapeutic efficacy and leads to an overall 5-year survival of 50-70% (9). In recent years, an increasing number of potential prognostic indicators in clinical practice have been discovered to predict the postoperative prognosis of patients with liver cancer (10–12). However, these indicators are often limited to traditional clinicopathological parameters or the composite scores of peripheral inflammatory cells, such as neutrophils, lymphocytes, and platelets (10, 13, 14). Therefore, more comprehensive, easily accessible, and practical clinical prognostic indicators are required for predicting prognosis of postoperative HCC patients and help them improve the future outcome.

Inflammation, immunity, and nutrition status play important roles in tumorigenesis, progression, and metastasis (15, 16), and are widely considered as viable prognostic tools for clinical outcomes in HCC patients (10, 11, 13). Previous studies have clearly demonstrated that systemic inflammatory response biomarkers, such as neutrophil lymphocyte ratio (NLR), platelet lymphocyte ratio (PLR), systemic immune-inflammation index (SII), and systemic inflammation response index (SIRI) are reliable predictors for prognosis of HCC patients undergoing surgical treatment (10, 13, 14). High-sensitivity C-reactive protein (hsCRP) is the most sensitive protein synthesized by the liver to detect systemic inflammation, and it is closely related to the prognosis of HCC patients (15, 17, 18). In addition, serum albumin (ALB) is considered to be the simplest and effective factor reflecting human nutritional status and liver function, and is also the decisive factor of cancer cell immune response (5, 19).

In a recent study, the inflammation-immunity-nutrition score (IINS), which was constructed by the combination of hsCRP, lymphocyte (LYM), and ALB, has been proved to be a powerful prognostic predictor for overall survival (OS) in patients with colorectal cancer (20). However, whether IINS presents good prognostic performance in HCC patients who received radical resection has not been verified. Therefore, this study aimed to evaluate the prognostic value of IINS in HCC patients after radical surgery.

## Materials and methods

### Patients and study design

**Figure 1:**
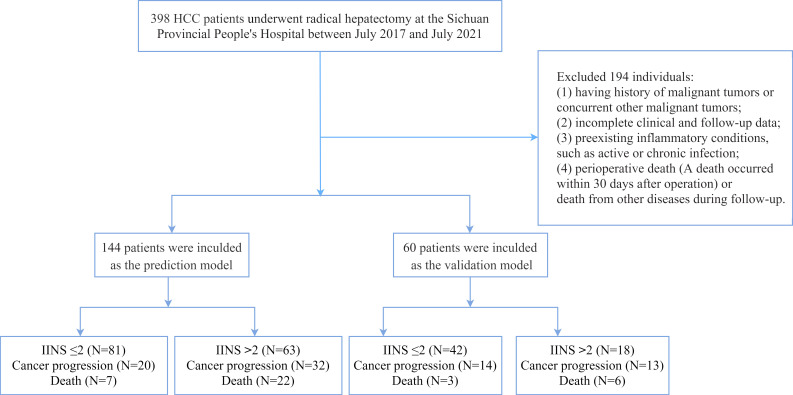
The flowchart of patients enrolled in this study. HCC, hepatocellular carcinoma; IINS, inflammation-immunity-nutrition score.

We retrospectively collected preoperative and postoperative clinicopathological data of HCC patients who received radical hepatectomy at the Sichuan Provincial People’s Hospital between July 2017 and July 2021. All serum indicators were collected within 1 week before the operation. The inclusion criteria are as follows (1): histopathological evaluation confirmed HCC (2); received radical resection (3); no preoperative therapy for primary HCC (4); initial diagnosis rather than recurrent tumors (5); hepatectomy with tumor-negative resection margins (6); Eastern Cooperative Oncology Group (ECOG) performance status 0–1. The exclusion criteria are as follows (1): having history of malignancies or concurrent with other malignancies (2); incomplete clinical and follow‐up data (3); having diseases of the hematologic system (4); preexisting autoimmune or systemic inflammatory disease (5); perioperative death (death within 30 days after surgery) or death from other diseases during follow-up. Pathological stage was confirmed according to the 7th American Joint Committee on Cancer staging. Finally, a total of 144 patients who were included from July 2017 to July 2021 were defined as the prediction model. The clinicopathological data of the other 60 patients were collected as the validation model for external validation. The flow chart of the study design is showed in **Figure 1**.

The experimental protocol was established, according to the ethical guidelines of the Helsinki Declaration and was approved by the Human Ethics Committee of Sichuan Academy of Medical Sciences and Sichuan Provincial People’s Hospital (NO2021-447). Written informed consent was obtained from individual or guardian participants.

### Definitions

According to the correlation between the three indicators and the patients’ OS, the optimal cut-off values of hsCRP, ALB, and LYM were determined using the X-tile software version 3.6.1 (https://medicine.yale.edu/lab/rimm/research/software/, Yale University School of Medicine, New Haven, CT) (21). According to 2 cut-offs, hsCRP was divided into the following three groups: score 0: ≤ 2.47 mg/L, score 1: > 2.47 mg/L and ≤ 11.33 mg/L, score 2: > 11.33mg/L. And LYM and ALB were grouped as follows: LYM (score 0: > 1.60×10^9^/L, score 1: > 0.84×10^9^/L and ≤ 1.60×10^9^/L, score 2: ≤ 0.84×10^9^/L); ALB (score 0: > 39.8 g/L, score 1: > 35.2 g/L and ≤ 39.8 g/L, score 2: ≤ 35.2g/L). Then, the scores for hsCRP, LYM, and ALB were summed to obtain the inflammation-immunity-nutrition score (IINS). Since the median IINS was 2 in this study, IINS > 2 was defined as high IINS group.

To compare the prognostic valve of IINS with other predictors, we also investigated the prognostic performance of Child-Pugh grade, Barcelona Clinic Liver Cancer (BCLC) stage, alpha-fetoprotein (AFP), carcinoembryonic antigen (CEA), LYM, ALB, NLR, PLR, SII and SIRI for each patient. The optimum cut-off values for NLR, PLR, AFP, CEA, LYM, SII and SIRI derived from receiver operating characteristic (ROC) curves are presented in **Table 4** and **Table 5**. The NLR and PLR were defined as follows: NLR = neutrophil/lymphocyte counts and PLR = platelet/lymphocyte counts (13), whereas the SII was defined as platelet × neutrophil/lymphocyte counts (10). The SIRI was calculated by using the Qi’s original formula: SIRI= monocytes × neutrophil/lymphocyte (22).

### Follow up

After the surgery, survival data were obtained by outpatient visits and telephone follow-up every 3 months in the first year, and then every 6 months thereafter if there was no recurrence or metastasis. The primary outcome was overall survival (OS), which was counted as the interval from the date of curative surgery to the date of death, lost to follow-up, or the end of the follow-up (August 2021), whichever came first. And the secondary outcome was progression-free survival (PFS), which was defined as the interval from the date of curative surgery to the date of death, recurrence or metastasis, loss to follow-up, or the end of the follow-up (August 2021), whichever came first. Cancer progression was defined as tumor recurrence, metastasis, or death.

### Statistical analysis

The associations between the IINS and the clinicopathologic characteristics were analyzed using Pearson’s χ2 test or Fisher’s exact test. Continuous data were expressed as mean ± SD. The area under the ROC curve (AUC) and optimal cutoff value that were calculated by predicting the OS and PFS for the NLR, PLR, AFP, CEA, LYM, SII, SIRI, and PNI are shown in **Table 4** and **Table 5**. Survival curves were presented using the Kaplan–Meier method and the differences were compared by log rank test. Univariate and multivariate analyses were performed using the Cox proportional hazards model. Moreover, time-dependent ROC curves were used to detect the prognostic performance of IINS, AFP and IINS-AFP classification for OS, respectively. Compared with ordinary ROC curve, time-dependent ROC curve could observe the prognostic performance of indicators at specific time points after surgery (20). The validation of the prediction model includes internal validation and external validation. In the internal validation, the bootstrap method was used to test the internal validity of the prediction model. The clinicopathological data of 60 HCC patients were used as the external validation data. The C-Statistics representing the discrimination ability and the Hosmer-Lemeshow test (H-L test) representing the calibration ability were calculated separately. Two-tailed P < 0.05 was considered statistically significant. All statistical analyses were performed using SPSS statistical software (version 26; SPSS-IBM, Chicago, IL, USA) and GraphPad Prism version 9.2.0 (GraphPad, CA, USA).

## Results

### Patient characteristics

The baseline characteristics of all the 144 participants in the prediction model are shown in **Table 1**.** **A total of 123 (85.4%) patients were male, and the average age was 58 ± 12 years. 104 (72.2%) of them had hepatitis B virus infection, and their mean body mass index (BMI) was 22.66 ± 2.81. The mean SII and SIRI were 447.38 ± 410.79 and 1.67 ± 2.16, respectively. Child-Pugh grade A, B, C accounted for 96 (66.7%), 47 (32.6%), 1 (0.7%), respectively. The proportion of patients with BCLC stages of 0/A, B, and C were 52.8%, 15.3%, and 31.9%, respectively. A total of 118 (81.9%) patients had medium-high differentiation cancer. Microvascular invasion was noted in 51 (35.4%) patients and cirrhosis in 103 (71.5%) patients. After surgery, 75 (52.1%) patients underwent adjuvant transarterial chemoembolization (TACE). The median follow-up time of the study was 17 ± 10 months. At the end of follow-up, 52 (36.1%) presented cancer progression, and 29 (20.1%) patients died. Patients with high IINS were more likely to present cancer progression (61.5%) and death (75.9%).

**Table 1:** Associations of the IINS with the clinicopathologic characteristics of HCC patients in the prediction model.

IINS value (0–6)				
Characteristics	Overall (n = 144)	IINS≤2 (n=81)	IINS>2 (n=63)	P value
Gender				**0.013**
Male	123 (85.4%)	71 (57.7%)	52 (42.3%)	
Female	21 (14.6%)	10 (47.6%)	11 (52.4%)	
Age, years	58 ± 12	59 ± 11	55 ± 13	0.089
BMI, kg/m^2^	22.66 ± 2.81	22.63 ± 2.73	22.70 ± 2.93	0.844
Etiology of HCC				0.851
HBV	104 (72.2%)	59 (56.7%)	45 (43.3%)	
Others	40 (27.7%)	22 (55.0%)	18 (45.0%)	
ALB, g/L	36.96 ± 4.75	39.32 ± 3.80	33.92 ± 4.10	**<0.001**
LYM, 10^9^/L	1.62 ± 1.18	1.71 ± 0.73	1.51 ± 1.59	**<0.001**
hsCRP, mg/L	11.35 ± 21.96	2.76 ± 5.03	22.40 ± 29.32	**<0.001**
AFP, ng/mL	2019.93 ± 5180.67	923.23 ±3257.38	3429.96 ± 6679.91	0.175
CEA, ng/mL	13.85 ± 127.65	3.75 ± 9.75	26.84 ± 192.75	0.220
Child-Pugh grade				**<0.001**
A	96 (66.7%)	71 (74.0%)	25 (26.0%)	
B	47 (32.6%)	10 (21.3%)	37 (78.7%)	
C	1 (0.7%)	0 (0%)	1 (100.0%)	
BCLC stage				**0.001**
0/A	76 (52.8%)	52 (68.4%)	24 (31.6%)	
B	22 (15.3%)	12 (54.5%)	10 (45.6%)	
C	46 (31.9%)	17 (37.0%)	29 (63.0%)	
Microvascular invasion				0.553
No	93 (64.6%)	54 (58.1%)	39 (41.9%)	
Yes	51 (35.4%)	27 (52.9%)	24 (47.1%)	
NLR	2.88 ± 2.06	2.42 ± 1.91	3.48 ± 2.11	**<0.001**
PLR	114.37 ± 66.20	95.47 ± 43.19	138.67 ± 81.46	**0.002**
SII	447.38 ± 410.79	357.85 ± 265.98	562.49 ± 523.44	0.100
SIRI	1.67 ± 2.16	1.44 ± 4.60	1.97 ± 2.16	**0.030**
Histopathological type				0.870
Poorly differentiation	26 (18.1%)	15 (57.7%)	11 (42.3%)	
Medium‐high differentiation	118 (81.9%)	66 (55.9%)	52 (36.1%)	
Tumor number				**0.031**
Single	94 (65.3%)	59 (62.8%)	35 (37.2%)	
Multiple	50 (34.7%)	22 (44.0%)	28 (56.0%)	
Cirrhosis				0.471
No	41 (28.5%)	25 (61.0%)	16 (39.0%)	
Yes	103 (71.5%)	56 (54.4%)	47 (32.6%)	
Postoperative adjuvant TACE No Yes	69 (47.9%) 75 (52.1%)	40 (58.0%) 41 (54.7%)	29 (42.0%) 34 (45.3%)	0.691
Cancer progression				**0.001**
No	92 (63.9%)	61 (66.3%)	31 (33.7%)	
Yes	52 (36.1%)	20 (38.5%)	32 (61.5%)	
Death				**<0.001**
No	115 (79.9%)	74 (64.3%)	41 (35.7%)	
Yes	29 (20.1%)	7 (24.1%)	22 (75.9%)	

BMI, Body Mass Index; HCC, hepatocellular carcinoma; HBV, hepatitis B virus; BCLC, Barcelona Clinic Liver Cancer; ALB, albumin; LYM, lymphocyte; hsCRP, high sensitivity C-reactive protein; AFP, alpha-fetoprotein; CEA, carcinoembryonic antigen; NLR, neutrophil–lymphocyte ratio; PLR, platelet–lymphocyte ratio; SII, systemic immune-inflammation index; SIRI, systemic inflammation response index; IINS, inflammation-immunity-nutrition score. Bold values means the P value is significant.

### Relationships between the inflammation-immunity-nutrition score and clinicopathologic characteristics

A total of 81 patients had low IINS, whereas 63 patients had high IINS. The relationship between clinicopathological factors and IINS are presented in **Table 1**. The patients with high IINS were more likely to have higher BCLC stage (P = 0.001), Child-Pugh grade (P < 0.001), NLR (P < 0.001), PLR (P = 0.002), SIRI (P = 0.030) and hsCRP levels (P < 0.001), more tumor numbers (P = 0.031), but lower ALB (P < 0.001) and LYM (P < 0.001) levels. All differences were statistically significant (P < 0.05). However, IINS was not associated with age, BMI, microvascular invasion, histopathological type, or cirrhosis.

### Univariate and multivariate analyses of OS and PFS among the HCC patients

Univariate and multivariate analyses were performed for Child-Pugh grade, BCLC Stage, tumor number, microvascular invasion, AFP, ALB, IINS, and other clinicopathologic variables. Univariate Cox regression analysis revealed that BCLC Stage (P < 0.001), microvascular invasion (P = 0.008), AFP (P = 0.002), and IINS (P = 0.001) were all significantly associated with OS in HCC patients (**Table 2**). Moreover, BCLC Stage (P < 0.001), tumor number (P = 0.024), microvascular invasion (P < 0.001), and IINS (P = 0.005) had significant associations with PFS in HCC patients (**Table 3**). From the multivariate Cox regression analysis, we found that BCLC Stage (hazard ratio (HR): 5.077; 95% confidence interval (95% CI): 1.758-14.660; P = 0.003), AFP (HR: 2.692; 95% CI: 1.235-5.867; P = 0.013), and IINS (HR: 2.680; 95% CI: 1.113-6.449; P = 0.028) were significant prognostic markers for OS (**Table 2**). BCLC Stage (HR: 2.988; 95% CI: 1.311-6.807; P = 0.009), microvascular invasion (HR: 1.965; 95% CI: 1.069-3.610; P = 0.030), and IINS (HR: 1.874; 95% CI: 1.061-3.311; P = 0.031) emerged as the powerful prognostic factors of PFS (**Table 3**).

**Table 2:** Univariate and multivariate Cox regression analyses of the associations between the prognostic factors and the overall survival of the HCC patients.

Characteristics	Univariate Analysis			Multivariate Analysis	
	HR (95% CI)	P value		HR (95% CI)	P value
Child-Pugh grade (A vs. B-C)	1.557 (0.743-3.263)	0.241			
BCLC Stage (0-A vs. B-C)	6.629 (2.515-17.472)	**<0.001**		5.077 (1.758-14.660)	**0.003**
Tumor number (Single vs. Multiple)	1.978 (0.951-4.113)	0.068			
Microvascular invasion (No vs. Yes)	2.707 (1.292-5.670)	**0.008**		1.159 (0.519-2.590)	0.718
AFP, ng/mL (≤80.38 vs. >80.38)	3.288 (1.552-6.968)	**0.002**		2.692 (1.235-5.867)	**0.013**
BMI, kg/m2 (≤21.57 vs. >21.57)	0.554 (0.267-1.149)	0.113			
Cirrhosis (No vs. Yes)	1.063 (0.471-2.400)	0.883			
HBV infection (No vs. Yes)	1.647 (0.670-4.046)	0.277			
IINS (Low group vs. High group)	4.018 (1.716-9.408)	**0.001**		2.680 (1.113-6.449)	**0.028**

HCC, hepatocellular carcinoma; BCLC, Barcelona Clinic Liver Cancer; AFP, alpha-fetoprotein; BMI, Body Mass Index; IINS, inflammation-immunity-nutrition score. Bold values means the P value is significant.

**Table 3:** Univariate and multivariate Cox regression analyses of the associations between the prognostic factors and the progression-free survival of the HCC patients.

Characteristics	Univariate Analysis		Multivariate Analysis	
	HR (95% CI)	P value	HR (95% CI)	P value
Child-Pugh grade (A vs. B-C)	1.046 (0.591-1.854)	0.876		
BCLC Stage (0-A vs. B-C)	3.286 (1.819-5.935)	**<0.001**	2.988 (1.311-6.807)	**0.009**
Tumor number (Single vs. Multiple)	1.895 (1.088-3.303)	**0.024**	0.762 (0.373-1.577)	0.456
Microvascular invasion (No vs. Yes)	2.799 (1.597-4.905)	**<0.001**	1.965 (1.069-3.610)	**0.030**
AFP, ng/mL (≤80.38 vs. >80.38)	1.475 (0.850-2.560)	0.167		
BMI, kg/m2 (≤21.57 vs. >21.57)	0.734 (0.424-1.270)	0.269		
Cirrhosis (No vs. Yes)	0.775 (0.430-1.399)	0.398		
HBV infection (No vs. Yes)	1.295 (0.690-2.429)	0.421		
IINS (Low group vs. High group)	2.225 (1.272-3.891)	**0.005**	1.874 (1.061-3.311)	**0.031**

HCC, hepatocellular carcinoma; BCLC, Barcelona Clinic Liver Cancer; AFP, alpha-fetoprotein; BMI, Body Mass Index; IINS, inflammation-immunity-nutrition score. Bold values means the P value is significant.

### Prognostic analysis of the inflammation-immunity-nutrition score in HCC patients

**Figure 2:**
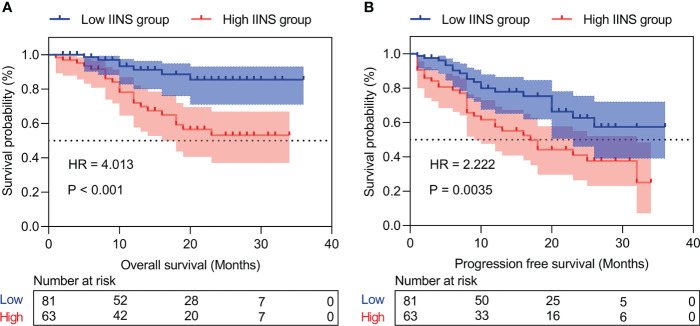
Kaplan‐Meier curves of inflammation-immunity-nutrition score (IINS) for overall survival **(A)** and progression-free survival **(B)**. IINS, inflammation-immunity-nutrition score.

**Figure 3:**
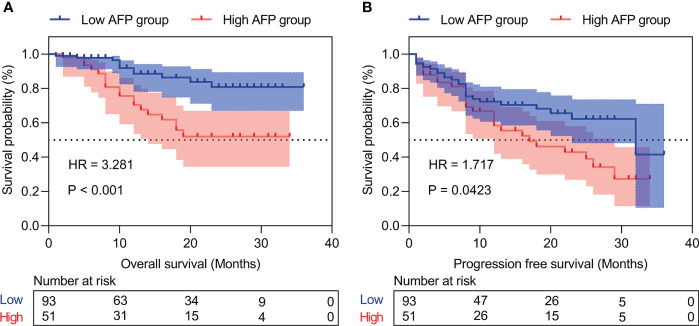
Kaplan‐Meier curves of the different AFP levels for overall survival **(A)** and progression-free survival **(B)**. AFP, alpha-fetoprotein.

**Figure 4:**
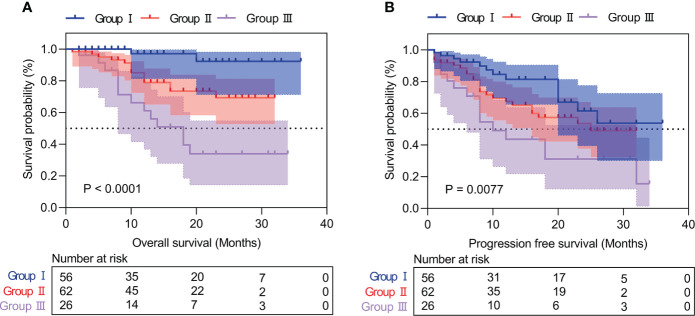
Kaplan‐Meier curves of IINS-AFP classification for overall survival **(A)** and progression-free survival **(B)**. IINS, inflammation-immunity-nutrition score; AFP, alpha-fetoprotein.

Patients with high IINS were associated with significantly worse OS and PFS compared with low IINS (HR: 4.013; 95% CI: 1.927-8.356; P < 0.001; **Figure 2A**; HR: 2.222; 95% CI: 1.281-3.855; P = 0.004; **Figure 2B**). Similar to IINS group, we found the OS and PFS of patients in the high AFP group (cutoff value = 80.38) were significantly worse than those in the low AFP group (HR: 3.281; 95% CI: 1.515-7.102; P < 0.001; **Figure 3A**; HR: 1.717; 95% CI: 0.980-3.009; P = 0.042; **Figure 3B**). Thus, IINS-AFP classification was formed by classifying patients using cut-off values of AFP and different IINS groups, and then dividing them into the following three groups: patients in low IINS group and with low AFP were group I, patients in high IINS group and with low AFP or in low IINS group and with high AFP were group II, patients in high IINS group and with high AFP were group III. We found that group I had a longer OS (Chi square: 24.03, P < 0.001, **Figure 4A**) and PFS (Chi square: 9.741, P = 0.008, **Figure 4B**) compared with group II and III.

### ROC analysis of the characteristics in HCC patients

**Figure 5:**
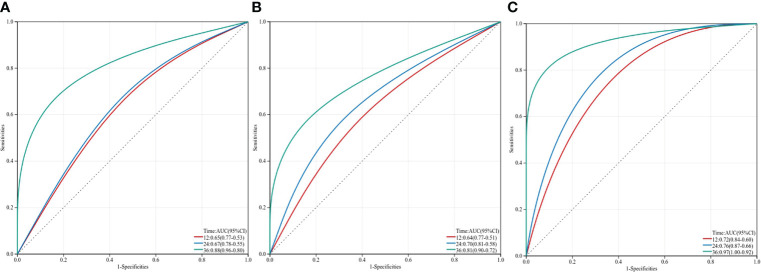
Three-year time-dependent ROC curves for overall survival of inflammation-immunity-nutrition score, AFP and IINS-AFP classification. IINS, inflammation-immunity-nutrition score; AFP, alpha-fetoprotein.

In our research, ROC analysis was used to evaluate the effect of different independent factors on prognosis. The results showed that the IINS-AFP classification (AUC: 0.767; 95% CI: 0.675-0.858; **Table 4**) was more predictive of OS in HCC patients than IINS (AUC: 0.760; 95% CI: 0.668-0.851; **Table 4**), although the difference was not statistically significant (P = 0.875). Moreover, the AUC of IINS-AFP classification for predicting OS was significantly higher than that of preoperative serum AFP levels (AUC: 0.668; 95% CI: 0.556-0.780; P = 0.043; **Table 5**) and other indicators. In terms of PFS, the AUC of IINS-AFP classification (AUC: 0.641; 95% CI: 0.546-0.735; P = 0.297; **Table 5**) was higher than that of other predictors, excluding its slightly lower predictive power than IINS (AUC: 0.679; 95% CI: 0.589-0.769; **Table 5**). In addition, the time-dependent ROC curves showed that both IINS (12-month AUC: 0.650, 24-month AUC: 0.670, 36-month AUC: 0.880) and IINS-AFP classification (12-month AUC: 0.720, 24-month AUC: 0.760, 36-month AUC: 0.970) performed well in predicting OS for HCC patients (**Figure 5**).

**Table 4:** Receiver operating characteristics analysis between IINS and other indicators in overall survival of the HCC patients.

Variables	Cut off value	AUC (95% CI)	Specificity	Sensitivity	P value
BCLC stage		0.751 (0.653-0.849)	0.617	0.828	**<0.001**
Child-Pugh grade		0.556 (0.435-0.677)	0.687	0.414	0.354
NLR	3.01	0.621 (0.500-0.743)	0.739	0.552	**0.044**
PLR	145.73	0.630 (0.502-0.758)	0.817	0.483	**0.031**
AFP	80.38	0.668 (0.556-0.780)	0.713	0.621	**0.005**
CEA	2.44	0.536 (0.451-0.620)	0.496	0.690	0.518
LYM	0.84	0.577 (0.492-0.659)	0.896	0.345	0.233
SII	517.02	0.621 (0.498-0.743)	0.791	0.483	**0.045**
SIRI	1.10	0.640 (0.520-0.759)	0.530	0.759	**0.020**
IINS		0.760 (0.668-0.851)	0.644	0.759	**<0.001**
IINS-AFP		0.767 (0.675-0.858)	0.470	0.931	**<0.001**

HCC, hepatocellular carcinoma; BCLC, Barcelona Clinic Liver Cancer; AFP, alpha-fetoprotein; CEA, carcinoembryonic antigen; LYM, lymphocyte; NLR, neutrophil–lymphocyte ratio; PLR, platelet–lymphocyte ratio; SII, systemic immune-inflammation index; SIRI, systemic inflammation response index; IINS, inflammation-immunity-nutrition score. Bold values means the P value is significant.

**Table 5:** Receiver operating characteristics analysis between IINS and other indicators in progression-free survival of the HCC patients.

Variables	Cut off value	AUC (95% CI)	Specificity	Sensitivity	P value
BCLC stage		0.674 (0.581-0.767)	0.644	0.667	**0.001**
Child-Pugh grade		0.513 (0.414-0.612)	0.687	0.414	0.793
NLR	2.59	0.664 (0.570-0.759)	0.733	0.556	**0.001**
PLR	109.9	0.614 (0.519-0.709)	0.633	0.537	**0.023**
AFP	305.21	0.549 (0.449-0.650)	0.778	0.352	0.328
CEA	2.44	0.566 (0.481-0.648)	0.522	0.654	0.188
LYM	0.92	0.572 (0.487-0.654)	0.848	0.346	0.165
SII	501.24	0.640 (0.545-0.736)	0.822	0.426	**0.005**
SIRI	1.03	0.649 (0.553-0.744)	0.533	0.722	**0.003**
IINS		0.679 (0.589-0.769)	0.656	0.593	**<0.001**
IINS-AFP		0.641 (0.546-0.735)	0.456	0.722	**0.005**

HCC, hepatocellular carcinoma; BCLC, Barcelona Clinic Liver Cancer; AFP, alpha-fetoprotein; CEA, carcinoembryonic antigen; LYM, lymphocyte; NLR, neutrophil–lymphocyte ratio; PLR, platelet–lymphocyte ratio; SII, systemic immune-inflammation index; SIRI, systemic inflammation response index; IINS, inflammation-immunity-nutrition score. Bold values means the P value is significant.

### Validation and performance of the prediction model

To ensure the credibility of the prediction model, the performance of the model was quantified by assessing its discrimination and calibration abilities. The baseline characteristics of all the 60 participants in the validation model are shown in **Supplemental Table 1**. As shown in **Table 6**, the internal validation found the model to have strong internal consistency and moderate predictive validity for clinical prognosis. The predictive performances of the prediction model for OS and PFS as measured by the C-Statistics were good (C-Statistics = 0.759; C-Statistics = 0.668). The external validation showed that the C-Statistics of IINS in OS and PFS were 0.716 and 0.665, respectively, which also represented that IINS had strong discrimination ability and external applicability. In the Hosmer-Lemeshow test (H-L test), the P values for OS and PFS were 0.769 and 0.971, respectively, indicating a strong calibration ability of the model. Therefore, IINS had good predictive performance, strong internal validity and external applicability, and could be used to establish the prediction model for predicting postoperative survival and recurrence in patients with HCC.

**Table 6:** Validation and performance of the prediction model for overall survival and progression-free survival.

Overall Survival	Inflammation-Immunity-Nutrition Score	
	Internal validation^b^	External validation^c^
**C-Statistics (95% CI)**	0.759 (0.652-0.866)	0.716 (0.523-0.908)
**P value^a^**	0.769	
**Progression-free Survival**	**Internal validation^b^**	**External validation^c^**
**C-Statistics (95% CI)**	0.668 (0.559-0.776)	0.665 (0.523-0.807)
**P value^a^**	0.971	

^a^Hosmer-Lemeshow test (H-L test); ^b^the resampling sample size is 100; ^c^the number of patients was 60.

## Discussion

In this study, we found that IINS might serve as a robust prognostic score in HCC patients after hepatectomy. Our results showed that patients with low IINS had significantly better OS and PFS than those with high IINS, and IINS was an independent risk factor for the clinical outcomes of HCC patients. Further comparison revealed the better prognostic performance of IINS than other indicators, such as Child-Pugh grade, BCLC stage, AFP, CEA, neutrophil lymphocyte ratio (NLR), platelet lymphocyte ratio (PLR), systemic immune-inflammation index (SII), and systemic inflammation response index (SIRI). To the best of our knowledge, this retrospective study was the first to identify the predictive value of inflammation-immunity-nutrition score in HCC patients who received radical hepatectomy.

In recent years, a lot of studies have shown the prognostic values of prognostic biomarkers for cancer prognosis, and elevated NLR, PLR, SII, SIRI, and PLR are associated with poor OS or RFS of patients with liver cancer (5, 10, 13, 14). However, most of the current prognostic biomarkers are different combinations of the two indicators in serum detection, which cannot reflect the immune and nutritional functions of the body, and thus lead to inevitable bias and prediction inaccuracy (23). Therefore, the Inflammation-immunity-nutrition score, which is based on a combined score of preoperative hsCRP, LYM and ALB, has been proved to have good prognostic performance in resectable CRC for OS (20), and the predictive value of IINS for HCC may also be explained by the role of these indicators. High IINS usually results from lymphopenia, hypoproteinemia, and increased hsCRP, suggesting high inflammatory response and low immune and nutritional status. Systemic inflammation is closely related to the proliferation, invasion and metastasis of malignant tumors (16, 24, 25), in which immune and nutritional status are important components of the inflammatory response (5). Furthermore, most liver cancers occur in chronically inflamed cirrhotic livers, this creates a pro-inflammatory environment that also promotes tumor formation and progression (25–28). Accumulating evidence has also suggested that hsCRP/LYM, hsCRP/ALB, and prognostic nutritional index (PNI) present powerful prognostic values in many types of cancer (5, 18, 19, 29). ALB is often used to assess liver function in patients with liver cancer, and hypoalbuminemia could reduce the systemic immune system, leading to tumor cell proliferation (5). Lymphocytes serve as the basis of cell-mediated anti-tumor immune responses, which could inhibit tumor cell proliferation and metastasis. Low lymphocyte counts could reduce the immune surveillance of cancer and lead to poor prognosis in various malignancies (30–32). As a result, IINS could be a practical, effective, and easily accessible clinical prognostic indicator for patients with HCC.

As an oncofetal antigen and a diagnostic marker for HCC, AFP has long been recognized as a useful predictor of the prognosis of liver cancer (33, 34). High AFP levels are usually associated with larger tumors, poorly differentiated histopathological type, and worse survival (35). This may be explained by the relationship between high AFP levels and the role of VEGF and VEGFR-2 in angiogenesis and promoting the growth of various malignances, including HCC (36, 37). Therefore, AFP has been applied to various HCC prognostic scoring systems in clinical practice (12, 38).

Serum AFP level mainly reflects the pathological conditions and tumor activity, while IINS reflects the overall status of the patient, including inflammation, immune, and nutritional status. In the current study, we also innovatively combined the application of IINS and AFP, and then performed an individualized prediction of the postoperative prognosis of HCC patients. Our study revealed that IINS-AFP classification had a better prognostic performance for survival and recurrence than other predictors, excluding its slightly lower predictive power for PFS than IINS. According to prognosis, group I was good, group II was moderate, and group III was poor. For patients in group I or II, curative surgery rather than palliative resection should be the primary consideration in resectable cases. While for patients in group III, considering the high mortality and recurrence rates, surgeons should carefully evaluate whether they can implement aggressive surgical treatment. Early treatment such as TACE, targeted therapy, and immunotherapy may prolong the survival time and enhance the quality of life for them. Furthermore, IINS-AFP classification could also serve as a practical guidance to help patients adjust follow-up strategies and improve future outcomes.

This study also has several limitations. First, although the cutoff value of IINS derived from clinical reference value, it may vary in different studies due to different sample sizes and patient selection criteria. Second, this study included patients only from one center, and the sample size in the study was limited. Further multicenter, large-scale prospective studies are needed to validate our findings.

## Conclusion

Our study demonstrated that preoperative IINS could serve as a powerful prognostic predictor for HCC patients after radical surgery. Specifically, IINS-AFP classification presents better prognostic performance than IINS or AFP alone, which may provide a simple way to identify patients with poor prognosis and an opportunity to guide treatment and follow-up strategies to improve their prognosis.

## Data availability statement

The raw data supporting the conclusions of this article will be made available by the authors, without undue reservation.

## Ethics statement

The experimental protocol was established, according to the ethical guidelines of the Helsinki Declaration and was approved by the Human Ethics Committee of Sichuan Academy of Medical Sciences and Sichuan Provincial People’s Hospital (NO2021-447). Written informed consent to participate in this study was provided by the participants’ legal guardian/next of kin.

## Author contributions

YL, ZZ was responsible for study conception and design, data acquisition, data analysis and drafting and revision of the manuscript. XH, YS was responsible for study conception and design, data analysis and drafting and revision of the manuscript. DZ, CL, ZD, HZ, TF were responsible for data acquisition. JS was responsible for drafting and revision of the manuscript. All authors contributed to the article and approved the submitted version.

## Funding

This study was supported by Sichuan Province Science and Technology Support Program (No. 2018HH0062), and Sichuan Province Science and Technology Support Program (No. 2021YFH0187).

## Conflict of interest

The authors declare that the research was conducted in the absence of any commercial or financial relationships that could be construed as a potential conflict of interest.

## Publisher’s note

All claims expressed in this article are solely those of the authors and do not necessarily represent those of their affiliated organizations, or those of the publisher, the editors and the reviewers. Any product that may be evaluated in this article, or claim that may be made by its manufacturer, is not guaranteed or endorsed by the publisher.
