# Supplementary material for: The prognostic significance of inflammation-immunity-nutrition score on postoperative survival and recurrence in hepatocellular carcinoma patients
Source: Front Oncol. 2022 Aug 9;12:913731. doi: 10.3389/fonc.2022.913731 (PMC9396284; doi:10.3389/fonc.2022.913731)
Supplement: Supplementary file 1 [file Table_1.docx]

**Supplemental Table 1** Associations of the IINS with the clinicopathologic characteristics of HCC patients in the validation model.

|  |  | **IINS value (0–6)** | |  |
| --- | --- | --- | --- | --- |
| **Characteristics** | **Overall**  **(n = 60)** | **IINS≤2 (n=42)** | **IINS>2 (n=18)** | **P value** |
| Gender |  | | | 0.094 |
| Male | 49 (81.7%) | 32 (65.3%) | 17 (34.7%) |  |
| Female | 11 (18.3%) | 10 (90.9%) | 1 (9.1%) |  |
| Age, years | 57 ± 10 | 56 ± 11 | 57 ± 10 | 0.693 |
| ALB, g/L | 36.04 ± 4.33 | 37.48 ± 3.93 | 34.69 ± 3.30 | **＜0.001** |
| LYM, 10^9^/L | 1.20 ± 0.48 | 1.34 ± 0.43 | 0.87 ± 0.43 | **＜0.001** |
| hsCRP, mg/L | 25.48 ± 46.80 | 15.55± 34.85 | 48.65 ± 62.13 | **0.002** |
| AFP, ng/mL | 7566.02 ± 26619.19 | 7244.97 ±25889.47 | 8315.82 ± 29014.21 | 0.640 |
| Child-Pugh grade |  | | | 0.072 |
| A | 43 (71.7%) | 33 (76.7%) | 10 (23.3%) |  |
| B | 15 (25.0%) | 8 (53.3%) | 7 (46.7%) |  |
| C | 2 (3.3%) | 1 (50.0%) | 1 (50.0%) |  |
| BCLC stage |  | | | 0.293 |
| 0/A | 35 (52.8%) | 26 (68.4%) | 9 (31.6%) |  |
| B | 22 (15.3%) | 15 (54.5%) | 7 (45.6%) |  |
| C | 3 (31.9%) | 1 (37.0%) | 2 (63.0%) |  |
| Microvascular invasion |  | | | 0.232 |
| No | 40 (66.7%) | 30 (75.0%) | 10 (25.0%) |  |
| Yes | 20 (33.3%) | 12 (60.0%) | 8 (40.0%) |  |
| Tumor number |  | | | 0.954 |
| Single | 23 (38.3%) | 16 (69.6%) | 7 (30.4%) |  |
| Multiple | 37 (61.7%) | 26 (70.3%) | 11 (29.7%) |  |
| Cirrhosis |  | | | 0.735 |
| No | 28 (46.7%) | 19 (67.9%) | 9 (32.1%) |  |
| Yes | 32 (53.3%) | 23 (71.9%) | 9 (28.1%) |  |
| Postoperative adjuvant TACE  No  Yes | 35 (58.3%)  25 (41.7%) | 25 (71.4%)  17 (68.0%) | 10 (28.6%)  8 (32.0%) | 0.777 |
| Cancer progression |  | | | **0.006** |
| No | 33 (55.0%) | 28 (84.9%) | 5 (15.1%) |  |
| Yes | 27 (45.0%) | 14 (51.9%) | 13 (48.1%) |  |
| Death |  | | | **0.010** |
| No | 51 (79.9%) | 39 (76.5%) | 12 (23.5%) |  |
| Yes | 9 (20.1%) | 3 (33.3%) | 6 (66.7%) |  |

**Abbreviations:** HCC, hepatocellular carcinoma; BCLC, Barcelona Clinic Liver Cancer; ALB, albumin; LYM, lymphocyte; hsCRP, high sensitivity C-reactive protein; AFP, alpha-fetoprotein; IINS, inflammation-immunity-nutrition score.

**Note:** Bold values means the P value is significant.
